# Infant Feeding

**Published:** 1885-05

**Authors:** 


### Infant Feeding.

Infant mortality everywhere is closely connected with infant feeding. In Norway, and in the Faroe Islands, where the smallest mortality among infants exists, they are invariably breast-fed, while in Bavaria and Iceland, where the mortality among infants is greatest, they are chiefly fed on cows' milk and farinaceous foods.

It is a fact full of practical instruction that, during the Franco-German war, when, by the sufferings of starvation in Paris caused by the siege, the general mortality of the population was doubled, that of the infants was reduced forty per cent. by the mothers being compelled to suckle their babies instead of feeding them artificially.

In times of famine in various places, where mothers have been obliged to suckle their infants, even though themselves restricted to a scanty supply of food, and not always of a wholesome kind, the improved vitality of infants has been the invariable result, demonstrating that even poor breast food is superior to the best artificial feeding.

